# MicroRNA-223-3p downregulates the inflammatory response in preeclampsia placenta via targeting NLRP3

**DOI:** 10.1186/s12884-024-06371-9

**Published:** 2024-03-06

**Authors:** Xueqiong Liu, Zhiyue Li, Dan Lu

**Affiliations:** 1 Clinical Medical College of Yangzhou University, Yangzhou, 225001 China; 2 The Second Affiliated Hospital of Xuzhou Medical University, Xuzhou, 221006 China

**Keywords:** Preeclampsia, Placenta inflammatory dysregulation, NLRP3 inflammasome, microRNA-223-3p

## Abstract

**Objective:**

To investigate the regulatory role of miR-223-3p in the inflammatory response of PE placenta.

**Methods:**

PE and normal placental tissues were collected to measure the expression of NLRP3 and miR-223-3p. The targeting relationship between NLRP3 and miR-223-3P was verified by bioinformatics analysis and classical double-luciferase reporter gene assay. Lipopolysaccharide (LPS) was used to induce HTR8/SVneo cells as PE placental cell inflammation model. Then we transfected miR-223-3p overexpression/miR-223-3p negative control plasmid into the LPS-induced HTR8/SVneo cells. Next, the expressions of NLRP3, Caspase-1, GSDMD, IL-1β and IL-18 were evaluated to elucidate the regulatory effect of miR-223-3p on the inflammatory response mediated by NLRP3 in PE placenta.

**Results:**

Compared with normal controls, NLRP3 was significantly up-regulated in PE placenta, while miR-223-3p was down-regulated. In addition, NLRP3 was a direct target of miR-223-3p. Further research revealed that the expression of NLRP3, Caspase-1, GSDMD, IL-1β and IL-18 could be obviously promoted in HTR8/SVneo cells treated with LPS (500 ng/ml) for 24 h, nevertheless it could be significantly suppressesed under the overexpression of miR-223-3p.

**Conclusion:**

MiR-223-3p suppressed NLRP3 inflamariomes activation, downstream inflammatory factors secretion and pyroptosis in LPS-induced HTR8/SVneo cells indicating that miR-223-3p could serve as an anti-inflammatory factor in preeclampsia.

**Supplementary Information:**

The online version contains supplementary material available at 10.1186/s12884-024-06371-9.

## Introduction

Preeclampsia (PE) is a syndrome specific to pregnancy accompanied with multi-organ and multi-system damage [1]. It is characterized by the new occurrence of hypertension and proteinuria after 20 weeks of gestation [2], affecting about 5-8% of pregnancies worldwide [3]. Furthermore, it is not only the main cause of maternal and perinatal morbidity and mortality [2], such as placental abruption, premature birth and fetal intrauterine growth restriction, but also an important risk factor for the increase of cardiovascular disease in mothers in later life [4, 5]. Although PE is caused by multiple factors, placental factors are the prerequisite for PE, among which placental inflammatory disorder is closely related to the pathogenesis of PE [6–9]. However, there is still no effective method to treat PE now, except to avoid the placenta, so we are eager to find an effective treatment for the PE placental inflammation.

Nod-like receptor pyrin domain-containing 3 (NLRP3) inflammasome, a multiprotein complexes comprised of NLRP3, apoptosis associated speck-like protein containing a CARD (ASC) and the effector protein precysteine hydrolase − 1 (pro-caspase-1), plays a vital role in the innate immune system [10]. It can be recognized and activated by a series of endogenous and exogenous pattern receptors, releasing mature IL-1β and IL-18, then participating in the regulation of various inflammatory diseases such as type 2 diabetes, multiple sclerosis and atherosclerosis [11, 12]. Previous studies have further demonstrated that the NLRP3 inflammasome was highly increased in placentas of PE, which suggested a close association between the activated NLRP3 inflammasome and the pathogenesis of PE [12–14]. Endogenous danger signals such as uric acid, adenosine triphosphate (ATP) and reactive oxygen species (ROS) can activate NLRP3 inflammasomes in placental trophoblasts, promoting the maturation and secretion of IL-1β and IL-18, even recruiting TNF-a, IL-33, IL -10 and other inflammatory cytokines to the materno-fetal interface to participate in the induction of inflammatory responses in the placenta [13, 15].

MicroRNA-223-3p (miR-223-3p) is a hematopoietic cell derived miRNA, who plays an important role in regulating monocyte-macrophage differentiation, neutrophil recruitment and pro-inflammatory response [16]. Some studies have shown that the absence of miR-223 leads to an enhanced inflammatory response, while overexpression of miR-223 reduces the pro-inflammatory response. According to the bioinformatics analysis, NLRP3 is a direct target gene of miR-223-3p [17, 18]. Furthermore, numerous researches have demonstrated that miR-223-3p can inhibit the inflammatory response in the inflammation-related diseases, such as rheumatic arthritis and Crohn’s disease, by targeting NLRP3 [19, 20]. In addition, miR-223 is localized in the trophoblast cells of placenta [21]. However, whether miR-223-3p regulates the activation of NLRP3 inflammasomes in PE placenta has not been reported yet. Therefore, this study aimed to clarify whether miR-223-3p can be a specific inhibitor of NLRP3 to suppress the inflammatory response in the placenta of PE, hoping to find a promising therapeutic approach for the treatment of PE.

## Materials and methods

### Ethics statement

This study was approved by the Ethics Committee of Subei People’s Hospital, Yangzhou City, Jiangsu Province. All tissue samples and data information from patients were obtained with the informed consent of participants before participating in this study.

### Study population characteristics

From December 2018 to January 2020, 60 pregnant women were hospitalized and delivered in the Subei People’s Hospital of Jiangsu Province, including 30 cases in the PE group (*n* = 30) and 30 cases in the normal control group (*n* = 30). The diagnostic criteria for PE included in the group were new systolic blood pressure ≥ 140 mmHg and/or diastolic blood pressure ≥ 90 mmHg after 20 weeks of pregnancy, accompanied by urine protein ≥ 0.3 g/24 h, or random urine protein ≥ (+), or anuria protein, but combined with any of the following: thrombocytopenia, liver damage, renal damage, pulmonary edema, or new central nervous system abnormalities or visual impairment. These symptoms were caused by preeclampsia, not another condition. All study subjects were singleton pregnancy, epidural anesthesia and cesarean section delivery, no previous history of acute or chronic diseases, no history of hypertension, diabetes, heart disease, endocrine or metabolic diseases, liver or kidney disease, no rheumatism, rheumatoid, erythema Lupus or other autoimmune-related diseases, no infections, inflammations, no adverse pregnancy outcomes (fetal growth restriction, premature delivery), no other pregnancy complications, no bad habits such as tobacco and alcohol.

### Sample collection and processing

Under aseptic conditions, three pieces of placenta about 1 cm × 1 cm × 1 cm in size from the center of maternal placenta near the umbilical cord were taken within 5 min after delivery, and abnormal areas such as infarct calcification and bleeding were avoided. After rinsing with sterile normal saline three times, two of them were put into the container containing RNA protection solution and formalin-fixed solution respectively and stored at 4 ℃. The other one was put into a sterile cryopreservation tube and stored in liquid nitrogen until the next use.

### Cell culture and treatment

HEK-293T cells were donated by Professor Yu Duonan from the Non-coding RNA Translational Medicine Laboratory of Yangzhou University. HTR8/SVneo cells were purchased from the Shanghai Mingzhou Company, which were derived from American Tissue Culture Collection (ATCC) passage cells. The two cell lines were cultured in DMEM (HyClone, Logan, UT, USA) and RPMI-1640 (HyClone, Logan, UT, USA) medium containing 10% fetal bovine serum (FBS, Gibco, USA), respectively. They were cultured in an incubator at 37 ℃ with 5% CO_2_. When the cell confluence reached 90%, the cells were passaged on. The cells in the logarithmic growth phase (the 3rd to 8th generation) were inoculated into a 24-well culture plate. After 24 h, the cells grew to a density of 60-80%, and Lipofectamine 2000 was used for transfection.

### Real time quantitative polymerase chain reaction (RT-qPCR)

Total RNA was isolated from placental tissues (100 mg/sample) and cells using Trizol reagent (Invitrogen). The concentration and purity of total RNA were determined by ultraviolet spectrophotometer, and the integrity of RNA was measured by agarose gel electrophoresis. Then the samples with an A260/A280 ratio of 1.8-2.0 and good RNA integrity were selected to synthesize the cDNA first strand. 0.5 ug RNA samples were reversely transcribed to cDNA using PrimeScriptTM RT Master Mix or Mir-XTM miRNA First-strand Synthesis Kit (Takara, Dalian, China), respectively. Finally, primers were designed and then synthesized by GenScript Biotechnology Ltd (Jiangsu, Nanjing, China), as shown in Table 1. Then the reaction liquid mixed according to the operating instructions of the SYBR Green Mastermix kit (Takara, Dalian, China) was performed the polymerase chain reaction on the biological real-time PCR system (Roche, LightCycler96). The reaction condition was that the initial denaturation step (95 °C for 30 s), the PCR reaction includes 40 cycles (95 °C for 5 s, 60 °C for 60 s, 95 °C for 10 s). U6 and glyceraldehyde-3-phosphate dehydrogenase (GAPDH) were used as internal references. Referring to the melting curve, the fold change of the relative expression of miR-223-3p and NLRP3 related genes were calculated by means of relative quantification (2- ΔΔCt method). The formula was as follows: ΔΔCT = ΔCT experimental group -ΔCT control group, whereΔCT = CT (target gene) - CT (internal reference). The experiment was repeated three times to obtain the average value.

**Table 1:** Primer sequences for RT-qPCR

Gene name	Sequence	
	Forward (5’-3’)	Reverse (5’-3’)
miR-223-3p	TGTCAGTTTGTCAAATACCCCA	universal primer
NLRP3	GAGGAAAAGGAAGGCCGACA	TGGCTGTTCACCAATCCATGA
ASC	TCACCGCTAACGTGCTG	TGGTCTATAAAGTGCAGGCC
Caspase-1	TTTCTGCTCTTCCACACCAG	AATGAAAATCGAACCTTGCGG
GSDMD	TGGTCTATAAAGTGCAGGCC	GACGTCCAAGTCAGAGTCAATAA
IL-1β	GGACAGGATATGGAGCAACAA	CCCAAGGCCACAGGTATTT
IL-18	CCAAGGAAATCGGCCTCTATT	CATACCTCTAGGCTGGCTATCT
U6	universal primer	universal primer
GAPDH	CGTGGAAGGACTCATGACCA	GGCAGGGATGATGTTCTGGA

RT-qPCR, reverse transcription quantitative polymerase chain reaction; miR-223-3p, microRNA-223-3p; NLRP3, Nod-like receptor pyrin domain-containing 3; ASC, apoptosis-associated speck-like protein containing a CARD; Caspase-1, the effector protein precysteine hydrolase − 1; GSDMD, gasdermin D; IL-, interleukin; GAPDH, glyceraldehyde-3-phosphate dehydrogenase.

### Western blot assay

The total protein was extracted from placental tissues and HTR-8/SVneo cells using RIPA lysate (Beyotime, China) (with PMSF-protease and phosphatase inhibitor added), and then the protein concentration was measured by the BCA assay kit (Beyotime, China) according to the instructions of manufacturer. The protein samples of 30ug per well were added to 10% of the twelve-alkyl sulfate-polyacrylamide gel electrophoresis (SDS-PAGE) slot to separate the protein and then electrotransferred onto the PVDF membranes. Next, the nonspecific site of the membranes were blocked with 5% skimmed milk for 1 h at room temperature and incubated overnight at 4 ℃ with first antibodies NLRP3 (1:1000, ab214185, Abcam), Caspase-1 (1:500, ab62698, Abcam), GSDMD (1:500, sc-81,868, Santa Cruz) and β-actin (1:1000, AF0003, Beyotime). On the second day, the membranes were incubated with the horseradish peroxidase (HRP) labeled secondary antibodies (1:1000, A0208/A0216, Beyotime) at room temperature for 1 h. Finally, under the action of ECL luminescent liquid, immunoreactive signals were detected by the Bio-Rad gel imaging system (MG8600 Thmorgan Biotechnology Co., Ltd., Beijing, China). The IPP6.0 software (Media Cybernetics, Singapore) was used to analyze the gray values and calculate the relative protein levels of NLRP3, Caspase-1 and GSDMD. The experiment was repeated at least three times.

### Immunohistochemical (IHC)

The placental tissues were embedded into the wax block, cut into 4 μm-thick slices, placed on the glass slide, baked continuously at 65 ℃ for 4 h, dewaxed with xylene, dehydrated with gradient ethanol and distilled water. Then the slides were put into a high-pressure cooker containing 0.01 M citrate repair solution (pH = 6.0) for water bath repair. Next, the slides were treated according to the instructions of the UItraSensitive^TM^SP (Mouse/Rabbit) IHC Kit (KIT − 9720, MXB). The tissues were added with a drop of reagent A and incubated for 10 min at room temperature to eliminate endogenous peroxidase activity, followed by three 0.1 M phosphate buffer saline (PBS) rinsing (3 min/time). Then the slides were added with a drop of non-immune animal serum (reagent B) for antibody blocking and incubated for 10 min at room temperature. After removing reagent B, the slides were added with two drops of diluted NLRP3 antibody (1:200, ab214185, Abcam), and incubated overnight at 4 ℃. On the following day, the slides were taken out, then rewarmed at 37 ℃ for 60 min, followed by 0.1 M PBS washing (3 min/time * 3). The slides were added with A drop of biotin-labeled secondary antibody (reagent C), and incubated at room temperature for 10 min, followed by PBS washing. The slides were further incubated with one drop of streptavidin-peroxidase solution (reagent D) at room temperature for 10 min, followed by 5 min washing under running water. Subsequently, the slides were added with two drops of diaminobenzidine (DAB) dye for color development. The reaction time was controlled under the microscope, the reaction was terminated in time, and the slides were repeatedly rinsed under tap water. Finally, the slides were re-stained with hematoxylin nucleus, differentiated with 1% hydrochloric acid alcohol for 5 s, and returned to blue with 1% ammonia solution for 2 min. The slides were dehydrated in gradient ethanol and transparently treated in xylene. At last, the film was sealed with neutral gum and read with the NIKON microscope.

### Dual-luciferase reporter gene assay

The bioinformatics websites such as Targetscan (http://www.targetscan.org/), miRWalk (http://zmf.umm.uni-heidelberg.de/apps/zmf/mirwalk2/) and miRBase (https://www.mirbase.org/) were used to predict the potential binding site sequence of miR-223-3p and NLRP3 3’untranslated region (3’UTR). The wild type (wt) and mutant type (mut) binding site gene fragments of NLRP3 3 ‘UTR were synthesized respectively, then subcloned into the GV272 basic plasmid vector, to generate NLRP3 3’UTR-wt and NLRP3 3 ‘UTR-mut luciferase vector. In each well, 0.5 ug firefly luciferase reporter plasmid, 0.4 ug miR-223-3p overexpression plasmid (or miR-223-3p negative control plasmid) and 0.1 ug sea kidney luciferase plasmid (internal reference standard) were co-transfected into survival condition good HEK-293T cells by lipo2000 (Invitrogen). After 48 hours of transfection on the 24-well cell culture plate, the cells were collected and lysed, centrifuged for 3-5min, then the supernatant was obtained. According to the instructions, luciferase activity was detected by double-luciferase assay kit (Promega) and double-luciferase report analysis system. The results were expressed by the relative light unit ratio of firefly and renilla luciferase. All the experiments were repeated three times.

### Enzyme-linked immunosorbent assay (ELISA)

The supernatant of cultured cells was collected carefully in a sterile tube and centrifuged at 3000 rpm for 20 min. According to the instructions of ELISA Kit (JYM0083Hu / JYM0092Hu, Wuhan), the secreted inflammatory factors IL-1β and IL-18 were detected, with the detection range of 3.5 pg/ml to200 pg/ ml. The absorbance (OD value) of each hole was measured at 450 nm wavelength, and the standard curve was drawn with the concentration of a standard substance as abscissa and OD value as ordinate. Then a linear regression equation was obtained, in which the correlation coefficient R value between the linear sample regression and the expected concentration was above 0.99. According to the standard curve, the concentrations of the inflammatory factors IL-1β and IL-18 were calculated. All experiments were repeated three times to improve accuracy.

### Statistical analysis

All dates of this study were expressed as mean ± standard deviation (SD). Graphpad Prism 8.0 software was used for data mapping and statistical analysis. The independent sample t-test was used to analyze the comparison between the two groups. One-way analysis of variance (ANOVA) was used to evaluate the comparison between the multiple groups. *P* < 0.05 was considered statistically significant.

## Results

### Characteristics of the study population

The demographic and clinical characteristics of PE pregnant women (*n* = 30) and normal pregnant women (*n* = 30) recruited in this study are shown in Table 2. The age of pregnant women in the two groups was similar (*P* > 0.05). The systolic blood pressure, diastolic blood pressure, 24 h proteinuria, body mass index and uric acid of PE patients were significantly higher than those of normal pregnant women (*P* < 0.05), while the gestational weeks at delivery and the weight of newborn delivered were significantly lower than those of normal pregnant women (*P* < 0.05). These clinical characteristics have an important relationship in the occurrence, development and severity of PE.

**Table 2:** Demographic and clinical characteristics of the study population

Characteristics	Normal (*n* = 30)	Preeclampsia (*n* = 30)	*p*-value
Maternal age (years)	27.2 ± 2.6	28.4 ± 2.7	> 0.05
Gestational age (weeks)	39.1 ± 1.2	36.1 ± 2.8	< 0.05
BMI at delivery (kg/m^2^)	26.6 ± 2.4	30.0 ± 2.7	< 0.05
Systolic blood pressure (mmHg)	120.1 ± 8.4	159.2 ± 11.1	< 0.05
Diastolic blood pressure (mmHg)	78.2 ± 5.9	105.8 ± 6.7	< 0.05
24-h urinary protein (g)	None	3.1 ± 1.7	< 0.05
ALT (U/L)	21.7 ± 6.6	23.1 ± 7.0	> 0.05
BUN (mmol/L)	5.4 ± 1.0	4.8 ± 1.2	> 0.05
Cr (umol/L)	74.5 ± 14.4	67.0 ± 14.7	> 0.05
UA (mg/dl)	226.4 ± 53.0	432.3 ± 91.7	< 0.05
PLT (*10^9^/L)	167.3 ± 24.0	164.3 ± 34.0	> 0.05
Neonatal weight (g)	3428.0 ± 392.2	3052.3 ± 514.8	< 0.05

BMI, body mass index; ALT, glutamic-pyruvic transaminase; BUN, blood urea nitrogen; Cr, creatinine; UA, uric acid; PLT, blood platelet.

Data were expressed as mean ± standard deviation (SD); All p values were calculated using a Student’s t-test.

### The expression of NLRP3 was markedly increased in the PE placenta tissues

**Fig. 1:**
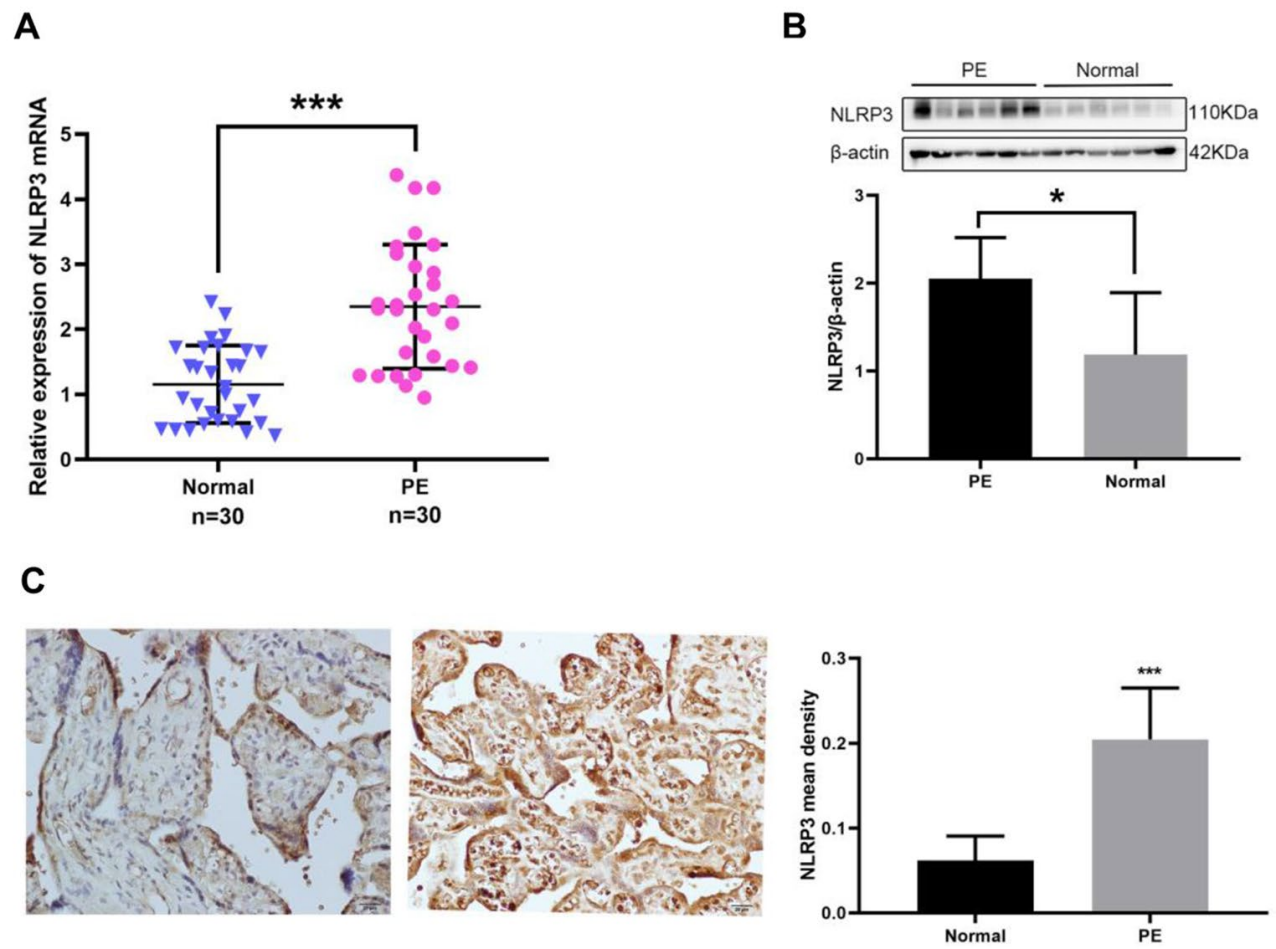
Expression patterns of NLRP3 in placenta tissues of patients with PE. (**A**) The expression of NLRP3 mRNA in PE and normal placenta tissues was detected by RT-qPCR; (**B**) The expression of NLRP3 protein in PE and normal placenta tissues was measured by western blot; (**C**) The positive protein expression level and location of NLRP3 in PE and normal placenta were determined by immunohistochemical staining (Bar = 20 μm). All data were displayed as mean ± SD; The data were analyzed by independent sample *t* test; *p < 0.05, ***p < 0.001 vs. the normal group; *N* = 30. PE, preeclampsia; NLRP3, Nod-like receptor pyrin domain-containing 3; RT-qPCR, real time quantitative polymerase chain reaction; SD, standard deviation

First, the expression of NLRP3 mRNA in the placenta tissues was detected by RTq-PCR assay. As shown in Fig. 1A, the expression of NLRP3 mRNA in the placenta tissues of PE patients (*n* = 30) was significantly upregulated compared to that of normal pregnancies (*n* = 30) (*P* < 0.001). To further confirm the expression pattern of NLRP3 in the placenta tissues of PE patients, Western blot and Immunohistochemical assay were used to detect the expression of NLRP3 at a protein level. The results showed that the protein expression level of NLRP3 in the placenta tissues of PE patients was significantly higher than that of normal pregnancy group (*P* < 0.05), and the NLRP3 positive protein was mainly expressed in the syncytial trophoblast cells, stromal cells and endothelial cells of the placenta, with light yellow, brown or tan appearance (Fig. 1B, C). This was consistent with the previous research results [6, 12], indicating that the upregulation of NLRP3 inflammasome is involved in the occurrence and development of PE.

### NLRP3 was a direct target of miR-223-3p, which was significantly downregulated in PE placenta tissues

**Fig. 2:**
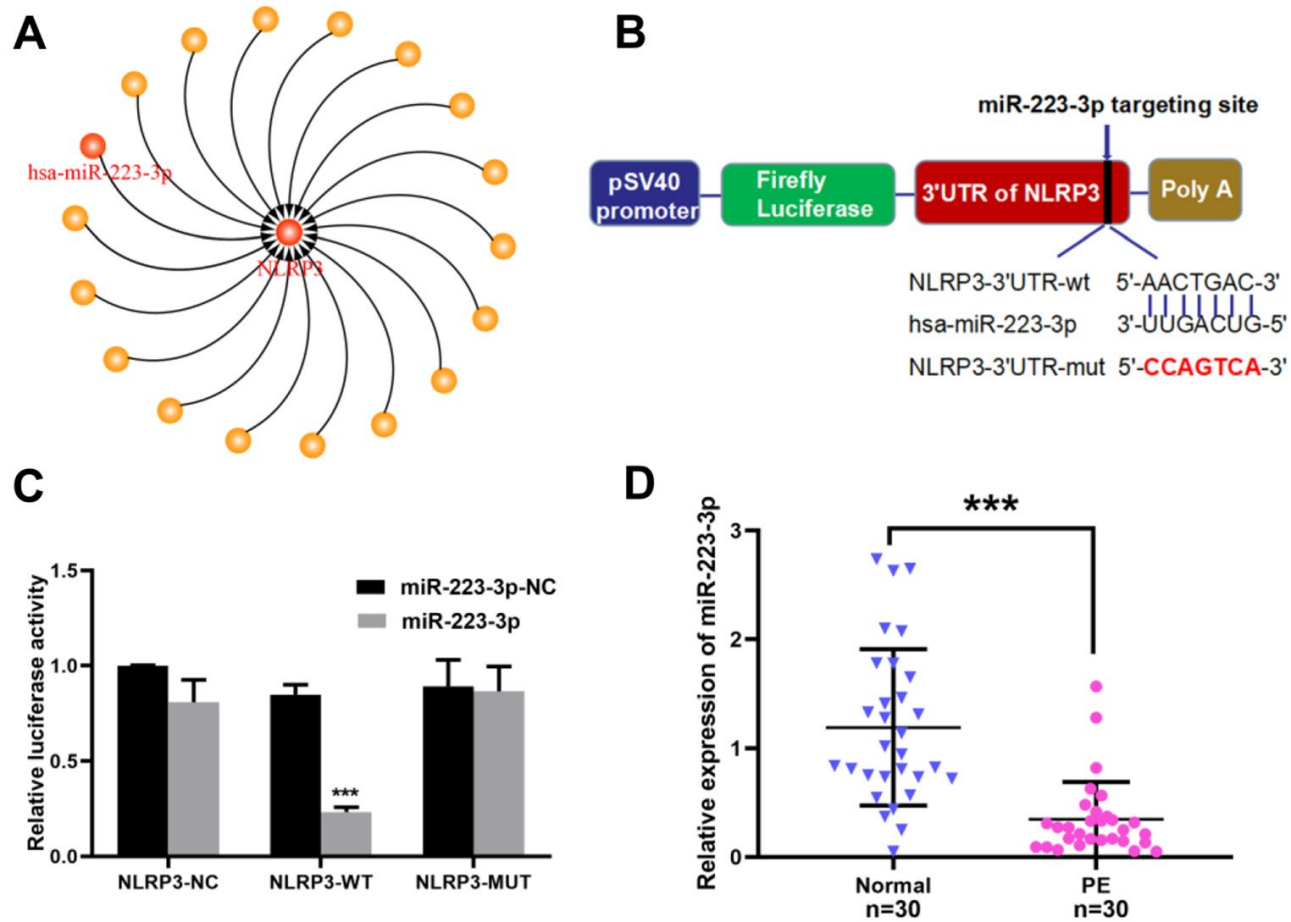
NLRP3 was a target gene of miR-223-3p, and miR-223-3p was downregulated in PE placenta tissues. (**A**) The upstream regulatory genes of NLRP3 were searched by TargetScan, miRWalk and miRBase Screen. (**B**) The binding site sequences between NLRP3-3’UTR-wt/ mut and miR-223-3p in the dual luciferase reporter gene vector were marked with a red typeface. (**C**) The luciferase activity of HEK-293T cells were detected by the dual-luciferase reporter gene assay after transfected the NLRP3-3’UTR-wt/ mut /negative control reporter plasmid and miR-223-3p overexpression/miR-223-3p negative control plasmid, as well as the internal reference Renilla plasmid into it using lipo2000. (**D**) The expression of miR-223-3p in PE and normal pregnancy placenta tissues was detected by RT-qPCR. All data were expressed as mean ± SD; In Fig. 2C, the data were analyzed by Two-way ANOVA (Tukey’s multiple comparisons test), *** *P* < 0.001 vs. the NLRP3-NC + miR-223-3p-NC group, the experiment was lindependently repeated three times; In Fig. 2D, the data were analyzed by the independent sample t test, *** *P* < 0.001 vs. the normal group; *N* = 30. PE, preeclampsia; NLRP3, Nod-like receptor pyrin domain-containing 3; miR-223-3p, microRNA-223-3p; WT, wild type; MUT, mutant type; NC, negative control; UTR, untranslated region; RT-qPCR, real time quantitative polymerase chain reaction; SD, standard deviation

Three major databases (Targetscan (http://www.targetscan.org/), miRWalk (http://zmf.umm.uni-heidelberg.de/apps/zmf/mirwalk2/) and miRBase (https://www.mirbase.org/) were used to explore the upstream regulators of NLRP3. The results showed that a large number of microRNAs had the role of targeting and regulating NLRP3. Among them, miR-223-3p, who had a specific binding site with NLRP3 3’UTR, was the most important gene closely related to inflammation and infection (Fig. 2A, B). Intriguingly, miR-223-3p had been reported in several studies that it could regulate the inflammatory diseases such as hepatitis [22], neuritis [23], and intestinal inflammation [20] by targeting the NLRP3 3’UTR region. Therefore, we speculated that miR-223-3p might participate in the regulation of inflammatory response in PE by targeting NLRP3. Subsequently, the classic dual-luciferase reporter gene experiment was used to verify the targeting relationship between miR-223-3p and NLRP3. As shown in Fig. 2C, the luciferase activity of HEK-293T cells transfected with NLRP3-3’UTR-wt and miR-223-3p overexpression plasmids was significantly decreased (*P* < 0.001), while it had no significant changes in cells transfected with NLRP3-3’UTR-mut and miR-223-3p overexpression plasmid (*P* > 0.05). In addition, the expression of miR-223-3p in the placent tissues was detected by RT-qPCR. The result showed that the expression of miR-223-3p in the placenta tissues with PE was significantly downregulated compared to the normal group(*P* < 0.05) (Fig. 2D). The above results indicated that the downregulation of miR-223-3p might be involved in the pathogenesis of PE by targeting and regulating the 3’UTR of NLRP3.

**3.4. Effects of lipopolysaccharide (LPS) stimulation at different concentrations on NLRP3-mediated inflammatory signaling pathways in HTR8/SVneo cells**.

**Fig. 3:**
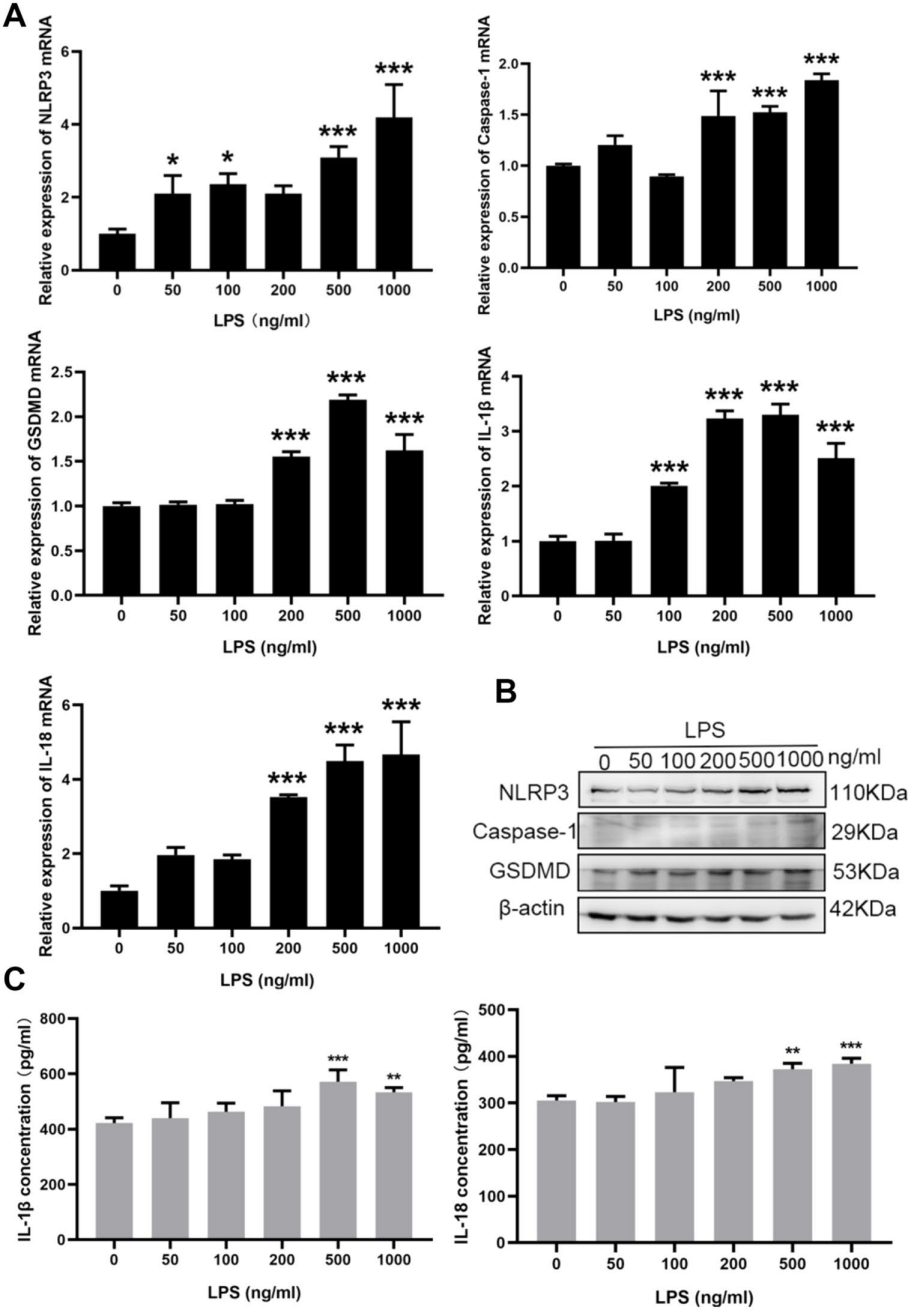
Expression of NLRP3 inflammasome-related molecules in HTR8/SVneo cells exposed to lipopolysaccharide (LPS). (**A**) The expression of NLRP3, Caspase-1, GSDMD, IL-1β, and IL-18 mRNA in HTR8/SVneo cells treated with different concentrations of LPS for 24 h was detected by RT-qPCR. (**B**) The protein expression of NLRP3, Caspase-1 and GSDMD in HTR8/SVneo cells was detected by western blot. (**C**) The expression of IL-1β and IL-18 in the culture supernatant of HTR8/SVneo cells was assessed by ELISA. All data were expressed as mean ± SD; The data were analyzed by Two-way ANOVA (Tukey’s multiple comparisons test), * *P* < 0.05; **P < 0.01; ***P < 0.001 vs. the blank group, the experiments were independently repeated three times. NLRP3, Nod-like receptor pyrin domain-containing 3; Caspase-1, the effector protein precysteine hydrolase − 1; GSDMD, gasdermin D; IL-, interleukin; LPS, lipopolysaccharide; RT-qPCR, real time quantitative polymerase chain reaction; ELISA, Enzyme-linked immunosorbent; SD, standard deviation

To establish the inflammatory model of PE placental cells, different concentrations of LPS were first used to induced HTR8/SVneo cells for 24 h in vitro. Then the mRNA expression of NLRP3 inflammasome-related molecules in HTR8/SVneo cells after LPS-induced was detected by RT-qPCR. The results showed that LPS could elevate the mRNA expression levels of NLRP3, Caspase-1, GSDMD, IL-1β, and IL-18 in HTR8/SVneo cells in a dose-dependent manner, of which the concentration of 500 ng/ml LPS stimulation was most significant (*P* < 0.001) (Fig. 3A). Next, the protein expression levels of NLRP3, Caspase-1 and pyroptosis associated protein GSDMD in HTR8/SVneo cells treated with different concentrations of LPS were detected by western blot assay. As shown in Fig. 3B, the expression of NLRP3, Caspase-1, GSDMD protein were significantly increased in HTR8/SVneo cells after LPS 500 ng/ml induced for 24 h (*P* < 0.001), which was correspond to the mRNA levels above. Subsequently, to further investigate the effect of LPS-induced HTR8/SVneo cells on inflammatory factors, the ELISA was used to detect the expression levels of IL-1β and IL-18 in the culture supernatant of HTR8/SVneo cells induced by different concentrations of LPS for 24 h. The results showed that the expression levels of IL-1β and IL-18 were significantly increased with LPS 500 ng/ml treated for 24 h (*P* < 0.001) (Fig. 3C). The results above indicated that LPS 500 ng/ml stimulated HTR8/SVneo cells for 24 h could significantly induce the activation of NLRP3 inflamposome in cells, release of downstream inflammatory factors, as well as pyroptosis of cells.

**3.5. MiR-223-3p could inhibit the activation of NLRP3 inflammasomes, pyrolysis and the release of downstream inflammatory factors in HTR8/SVneo cells induced by LPS**.

**Fig. 4:**
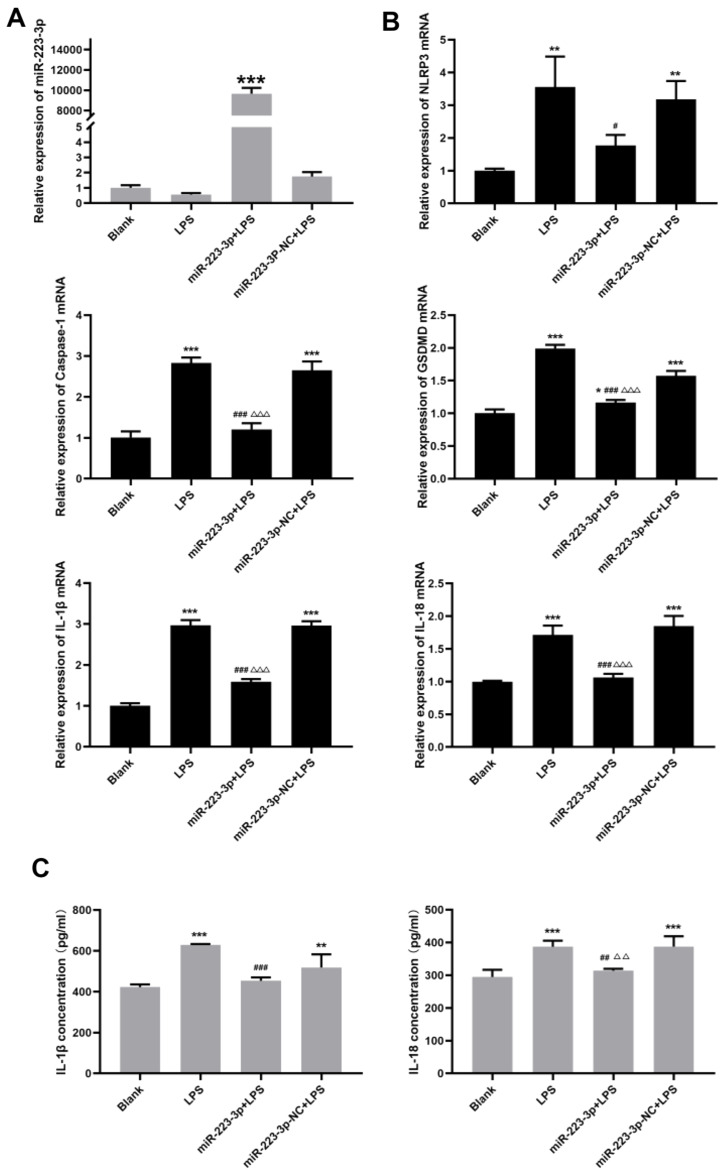
Effect of miR-223-3p on LPS-induced NLRP3 inflammasome mediated signaling pathway in HTR8/SVneo cells. (**A**) The HTR8/SVneo cells were transfected with miR-223-3p overexpression plasmids/miR-223-3p negative control plasmids for 48 h, then induced with LPS (500 ng/ml) for 24 h. RT-qPCR was performed to determine the expression level of miR-223-3p. (**B**) The expression of NLRP3, Caspase-1, GSDMD, IL-1β, and IL-18 mRNA in HTR8/SVneo cells with above treatment was detected by RT-qPCR. (**C**) The expression of IL-1β and IL-18 in the culture supernatant of HTR8/SVneo cells was assessed by ELISA. All data were expressed as mean ± SD; The data were analyzed by Two-way ANOVA (Tukey’s multiple comparisons test), *P < 0.05; ** *P* Δ 0.01; *** *P* Δ 0.001 vs. the blank group, # *P* Δ 0.05; ## *P* Δ 0.01; ### *P* Δ 0.001 vs. the LPS group; △*P* Δ 0.05; △△*P* Δ 0.01; △△△*P* Δ 0.001 vs. the miR-223-3p + LPS group, the experiments were independently repeated three times. miR-223-3p, microRNA-223-3p; NLRP3, Nod-like receptor pyrin domain-containing 3; Caspase-1, the effector protein precysteine hydrolase-1; GSDMD, gasdermin D; IL-, interleukin; LPS, lipopolysaccharide; RT-qPCR, real time quantitative polymerase chain reaction; ELISA, Enzyme-linked immunosorbent; SD, standard deviation

To evaluate the regulatory effect of miR-223-3p on LPS-induced NLRP3 inflammasome mediated signaling pathway in HTR8/SVneo cells, miR-223-3p overexpression plasmids/miR-223-3p negative control plasmids were transfected into HTR8/SVneo cells using lipo2000. After transfected for 48 h, cells were treated with LPS (500 ng/ml) for 24 h. Then the cells and culture medium supernatant were collected to extracte the RNA and protein for subsequent experiments. To investigate changes of miR-223-3p expression, RT-qPCR was performed to determine. As Fig. 4A showed, compared with the control group, the expression of miR-223-3p in the HTR8/SVneo cells with miR-223-3p overexpression plasmids transfected was significantly increased (*P* < 0.001), while the expression level of miR-223-3p in the miR-223-3p-NC group changed little. At the same time, RT-qPCR and western blot were used to detect the expression changes of NLRP3 inflammasome-related mRNA and protein in HTR8/SVneo cells after miR-223-3p overexpression and LPS induction. The results showed that overexpression of miR-223-3p can significantly inhibit the expression of NLRP3 inflammasome-related mRNA and protein induced by LPS (*P* < 0.001) (Fig. 4B). Subsequently, ELISA assay was used to evaluate the levels of downstream inflammatory factors in the NLRP3 inflammasomethe. The results showed that the overexpression of miR-223-3p significantly reduced the release of LPS-induced inflammatory factors IL-1β and IL-18 (*P* < 0.001) (Fig. 4C). These results indicated that overexpression of miR-223-3p could attenuate the activation of NLRP3 inflammasome, inhibit pyrolysis and the release of downstream inflammatory factors in LPS-induced HTR8/SVneo cells.

## Discussion

PE is a multifactorial and pathological disease peculiar to pregnancy. More and more studies [24–26] show that the excessive inflammatory reaction at maternal-fetal interface plays a crucial role in the pathogenesis of PE. NLRP3 inflammasome is an important mediator mediating the occurrence of immune inflammatory reaction on the maternal-fetal interface. What’s more, the expression of NLRP3 in PE placenta was markedly increased, which was closely related to the occurrence and development of PE [9, 12]. Previous studies have shown that miR-223-3p can specifically target the 3’UTR of NLRP3 and inhibit inflammatory reactions in inflammatory diseases such as rheumatoid arthritis, acute and chronic liver injury, tuberculosis [22, 27, 28]. Therefore, the purpose of this study was to explore whether miR-223 could be act as an inhibitor of NLRP3 to downregulate the inflammatory response in PE placenta.

First, we verified the high expression of NLRP3 in PE placenta by RT-qPCR, western blot, and Immunohistochemical experiments at the mRNA and protein levels, respectively, which was consistent with the previous descriptive research results of Weel and Liu et al [12, 13]. As the core protein of NLRP3 inflammasomes, the overexpression of NLRP3 on the maternal-fetal interface can over-activate NLRP3 inflammasomes, and then activate pro-Caspase-1 [29]. Activated Caspase-1 can lyse the inactive IL-1β and IL-18 precursors in cells, promote their maturation, secrete and release them into the blood. Then they can recruit other active inflammatory cytokines, chemokines, adhesion molecules, etc. to aggregate which can cause local placental inflammation, and even systemic inflammation, and then lead to extensive maternal endothelial cell dysfunction, reduce the invasiveness of trophoblast cells, and participate in the pathogenesis of PE. In addition, the activated Caspase-1 can also lyse the GSDMD protein in the cell, making the active GSDMD-N terminal domain accumulate on the cell membrane to form tiny holes, which can make the ion imbalance inside and outside the cell, causing trophoblast cell lysis and death accompanied by inflammation, which is the pyrosis of trophoblast [30]. Trophoblast cells subsequently expose to inflammatory stimulation inducing the secretion of pro-inflammatory cytokines, which can lead to trophoblast cell function defective, placental dysfunction and subsequent PE. Besides, Xu et al. and Pontillo et al. reported that specific NLRP3 gene polymorphism was closely associated with the significantly increased risk of PE [31, 32]. Therefore, the excessive activation of NLRP3 at the maternal-fetal interface plays an important role in the pathogenesis of PE. A large number of vitro and animal experimental studies have shown that NLRP3 inhibitors can effectively inhibit the inflammatory response, but there is still no specific therapeutic drug for PE, so searching for an effective inhibitor of NLRP3 may become a potential target for the treatment of PE.

MiR-223 is a microRNA closely related to inflammation and infection [17], consisting of miR-223-3p (guide strand) and miR-223-5p (auxiliary strand) [33], among which only the 3-arm (lead chain) of the precursor miR-223 (lead chain) is considered to be mature and function, while the complementary 5-arm (known as passenger chain) will be degraded [34]. A large number of researches have shown that miR-223 can specifically target the 3’UTR of NLRP3 to reduce the inflammation response. For example, overexpression of miR-223 in mouse neutrophils can reduce the activity of NLRP3 inflammasomes, which leads to decreased secretion of IL-1β, while miR-223 deficient mice can increase the level of NLRP3 in bone marrow derived neutrophils. In addition, studies have shown that the overexpression of miR-223 in human adenocarcinoma alveolar basal epithelial cells can reduce LPS-induced Caspase-1, IL-1β, and IL-18 levels by targeting NLRP3. However, as an anti-inflammatory protective agent, mir-223 has not been reported to strictly regulate the NLRP3-mediated inflammatory response in PE placenta at the molecular level. Therefore, we carried out a study based on this. First, we used RT-qPCR to detect the expression of miR-223-3p in PE placenta, finding that miR-223-3p was enriched in the placenta and expressed low in the PE placenta. Next, we used bioinformatics software and the classic dual-luciferase reporter gene experiment to preliminarily verify that NLRP3 is a direct target gene of miR-223-3p in HEK-239T cells.

Because LPS is a gram-negative bacterial endotoxin, whose lipids can induce innate immune responses mediated by NLRP3 inflammasomes. Ultra-low doses of LPS can induce specific persistent inflammatory state and PE-like clinical symptoms in pregnant rats, such as hypertension, proteinuria, thrombocytopenia, etc. Therefore, in this paper, the human transformed primary extravillous trophoblast cell line (HTR8/SVneo) was stimulated by LPS to establish a PE inflammatory cell model, and to explore whether miR-223 could regulate the inflammatory response in the PE placenta by targeting NLRP3. We first stimulated HTR8/SVneo cells with different concentrations of LPS and determined that 500 ng/ml LPS induced HTR8/SVneo cells for 24 h was an optimal condition, which can significantly promote the activation of NLRP3 inflammasomes, and the release of downstream inflammatory factors and the higher expression of GSDMD. Next, miR-223-3p overexpressed plasmids (or negative control plasmids) were transfected into HTR8/SVneo cells for 48 h and then treated with LPS (500 ng/ml) for 24 h to observe whether the overexpressed miR-223-3p could reverse the inflammatory response of HTR8/SVneo cells induced by LPS. Results showed that miR-223-3p was significantly highly expressed in HTR8/SVneo after transfection, and it could significantly reduce the activity of NLRP3 inflammasome induced by LPS and inhibit the secretion of inflammatory factors and pyrolysis.

MicroRNAs are stably expressed in the plasma. Interestingly, Zhang’s team has recently confirmed that absorbed plant miR-2911 in honeysuckle decoction can inhibit SARS-CoV-2 replication and accelerate the negative conversion of infected patients, revealing for the first time that exogenous miRNA can be directly absorbed and utilized by the animal’s gastric mucosa by oral administration, thus playing a post transcriptional regulating function [18]. This major new discovery suggests that miR-223-3p may become a specific inhibitor of NLRP3 in PE placental inflammation in the future.

**Fig. 5:**
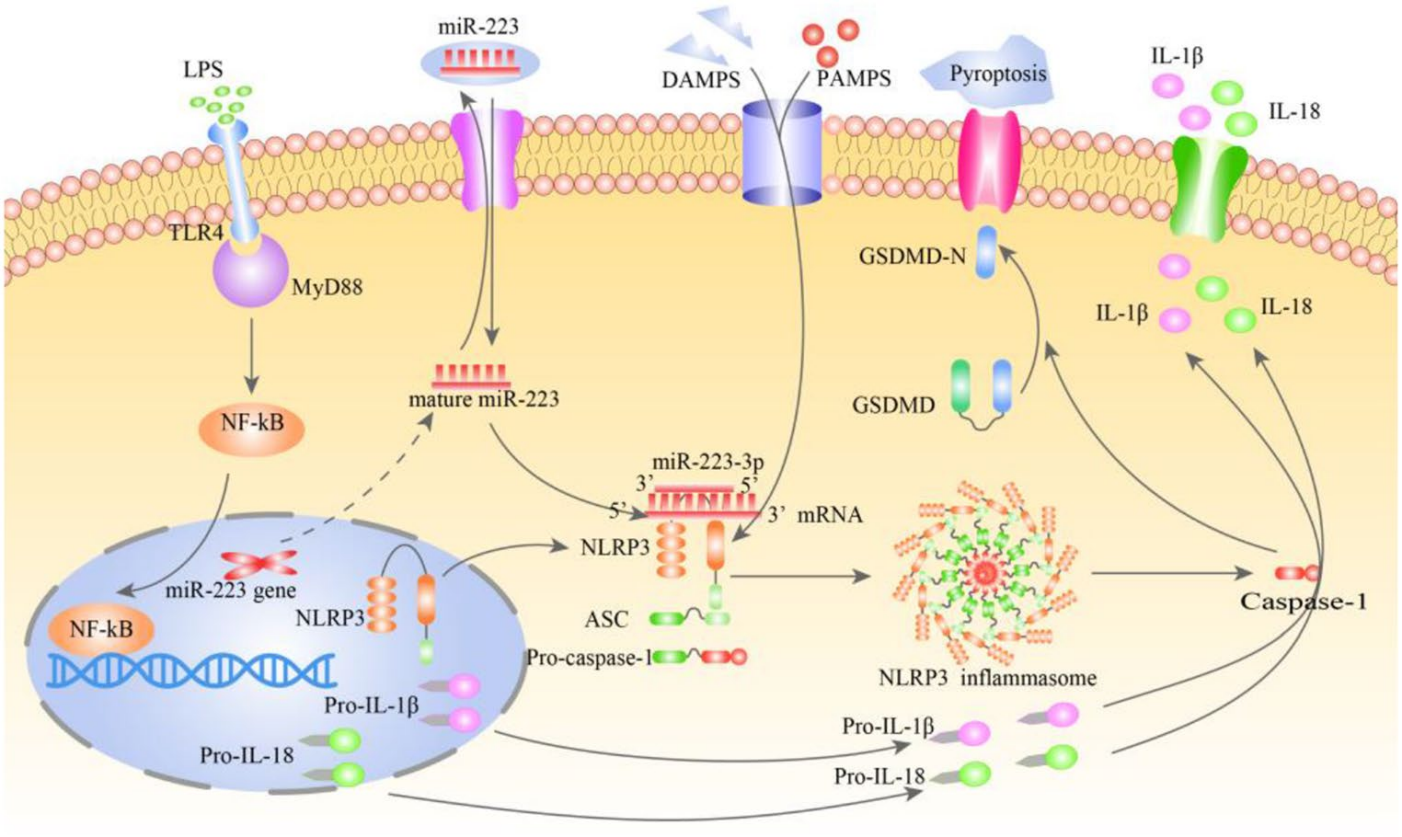
Schematic diagram illustrating the role of miR-223-3p on NLRP3 inflammasome signaling pathway in LPS-induced HTR8/SVneo cells. miR-223-3p inhibits the activation of NLRP3 inflammasomes, pyrolysis and the secretion of downstream inflammatory factors in LPS- induced HTR8/SVneo cells

In conclusion, our study demonstrated that miR-223-3p could inhibit the activation of NLRP3 inflammasomes, pyrolysis and the secretion of downstream inflammatory factors in LPS- induced HTR8/SVneo cells. The schematic diagram was shown in Fig. 5. This study provided a theoretical support for miR-223-3p to become a specific anti-inflammatory protective agent of NLRP3 in PE placenta, thus providing a new idea for the treatment of PE.

## Conclusion

MiR-223-3p suppressed NLRP3 inflamariomes activation, downstream inflammatory factors secretion and pyroptosis in LPS-induced HTR8/SVneo cells indicating that miR-223-3p could serve as an anti-inflammatory factor in preeclampsia.

### Electronic supplementary material

Below is the link to the electronic supplementary material.

**Supplementary Material 1:** Shows the original images of Fig.1B and Fig.3B

**Supplementary Material 2:** In our western blot assay, compared with the color pre-dyed maker bands, the PVDF membrane was cut into bands corresponding to the molecular weight of the target protein. These films were incubated overnight in the corresponding diluent of the primary antibody. Then they were fully washed and incubated in the second antibody. Once again, the films were fully washed, and finally the immunoreactive signals on the membrane were detected under the action of ECL luminescent liquid. We usually set the exposure time to 1s, 3s, 10s, 30s, 60s (the exposure time of the band with difficult luminescent was set to 120s). We generally saved such a luminous strip as 5 pictures, of which the first picture had the whitest background. The longer the exposure time, the darker the background color would be, and the more clearly you could see the edge of the film as well as the surrounding water traces, which you could refer to the picture provided in this Supplementary Material

**Supplementary Material 3:** All western blot images in Figure 1B and 3B we provided last time are original images (without any treatment). For a clearer view, you can refer to this Supplementary Material, which shows the membrane edges and water marks

## Data Availability

Data that supports the findings of this study are within the article. Further data is available from the corresponding author upon reasonable request.
