# Supplementary figures and images for: MicroRNA-223-3p downregulates the inflammatory response in preeclampsia placenta via targeting NLRP3
Source: BMC Pregnancy Childbirth. 2024 Mar 6;24:175. doi: 10.1186/s12884-024-06371-9 (PMC10918892; doi:10.1186/s12884-024-06371-9)

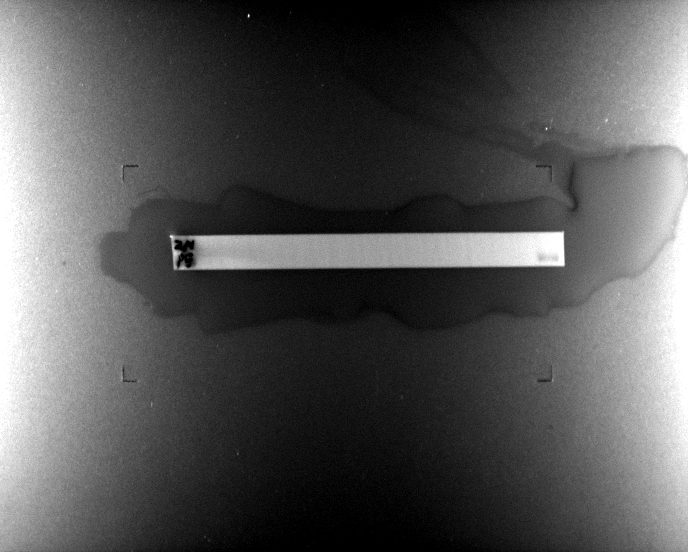


Fig.1B. NLRP3 stripe


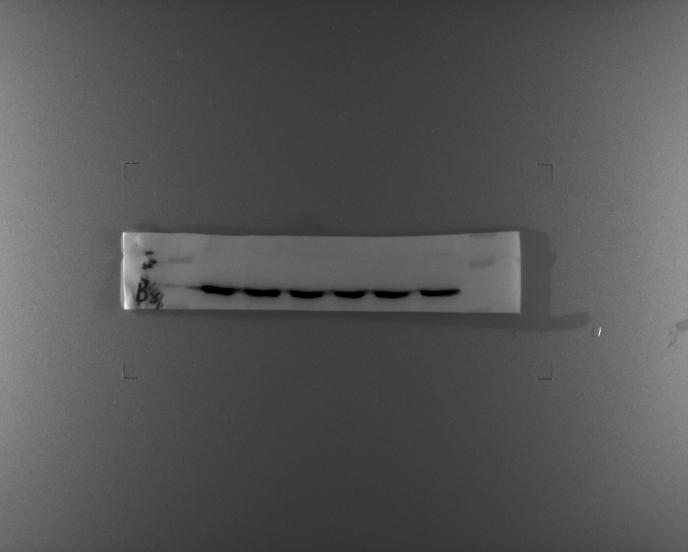


Fig.3B. β-actin and maker stripe


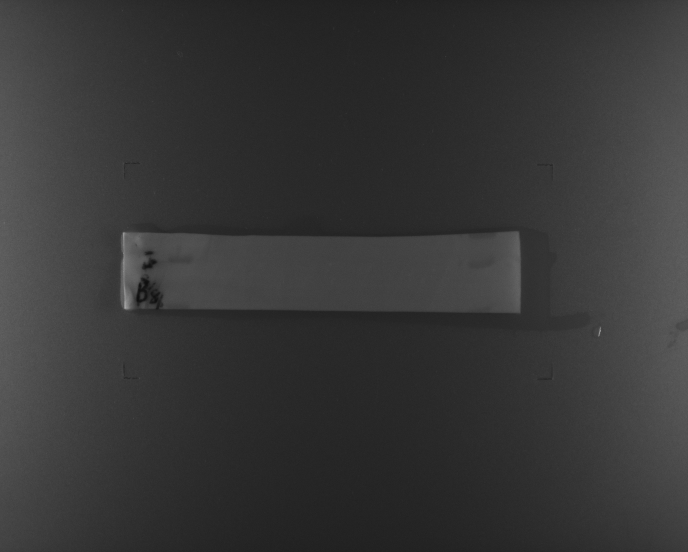


Fig.3B. β-actin stripe

Supplement: Supplementary file 2 — Supplementary Material 2: In our western blot assay, compared with the color pre-dyed maker bands, the PVDF membrane was cut into bands corresponding to the molecular weight of the target protein. These films were incubated overnight in the corresponding diluent of the primary antibody. Then they were fully washed and incubated in the second antibody. Once again, the films were fully washed, and finally the immunoreactive signals on the membrane were detected under the action of ECL luminescent liquid. We usually set the exposure time to 1s, 3s, 10s, 30s, 60s (the exposure time of the band with difficult luminescent was set to 120s). We generally saved such a luminous strip as 5 pictures, of which the first picture had the whitest background. The longer the exposure time, the darker the background color would be, and the more clearly you could see the edge of the film as well as the surrounding water traces, which you could refer to the picture provided in this Supplementary Material [file 12884_2024_6371_MOESM2_ESM.docx]
