# Supplementary material for: MicroRNA-223-3p downregulates the inflammatory response in preeclampsia placenta via targeting NLRP3
Source: BMC Pregnancy Childbirth. 2024 Mar 6;24:175. doi: 10.1186/s12884-024-06371-9 (PMC10918892; doi:10.1186/s12884-024-06371-9)
Supplement: Supplementary file 3 — Supplementary Material 3: All western blot images in Figure 1B and 3B we provided last time are original images (without any treatment). For a clearer view, you can refer to this Supplementary Material, which shows the membrane edges and water marks [file 12884_2024_6371_MOESM3_ESM.docx]

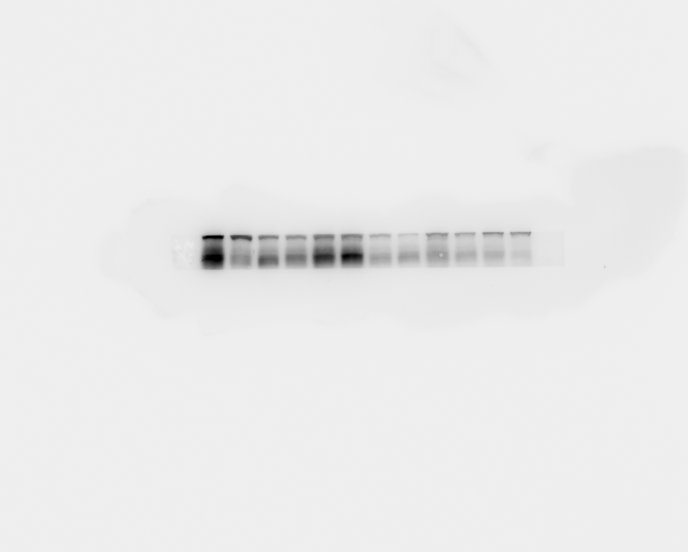


Fig. 1B. NLRP3 stripe


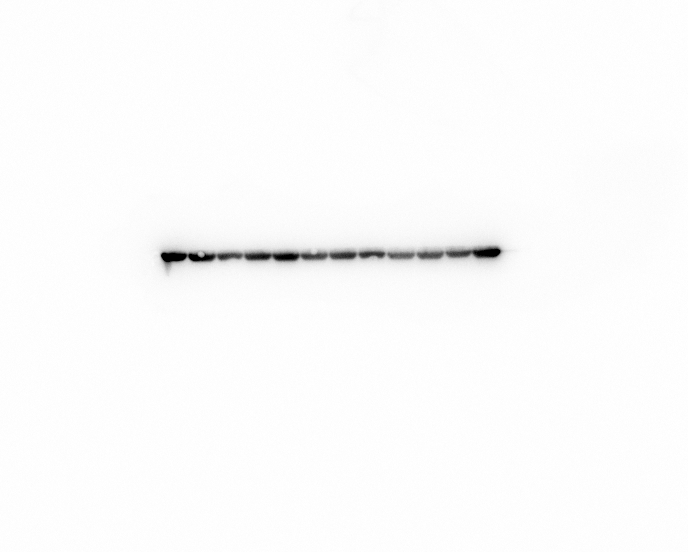


Fig. 1B. β-actin stripe


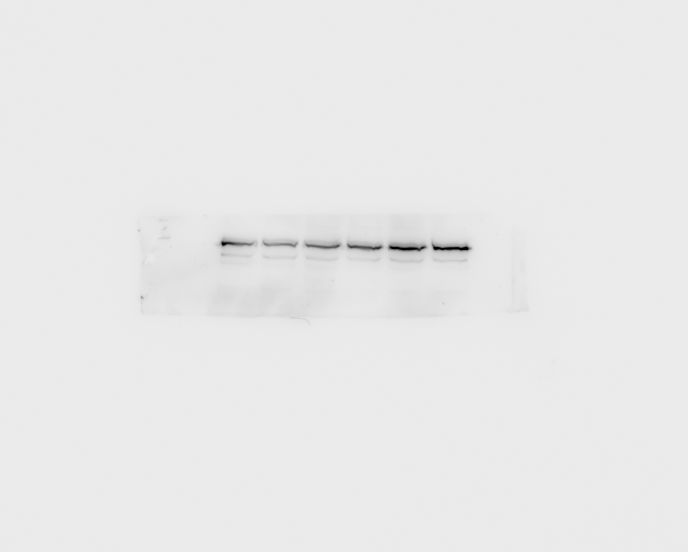


Fig. 3B. NLRP3 stripe


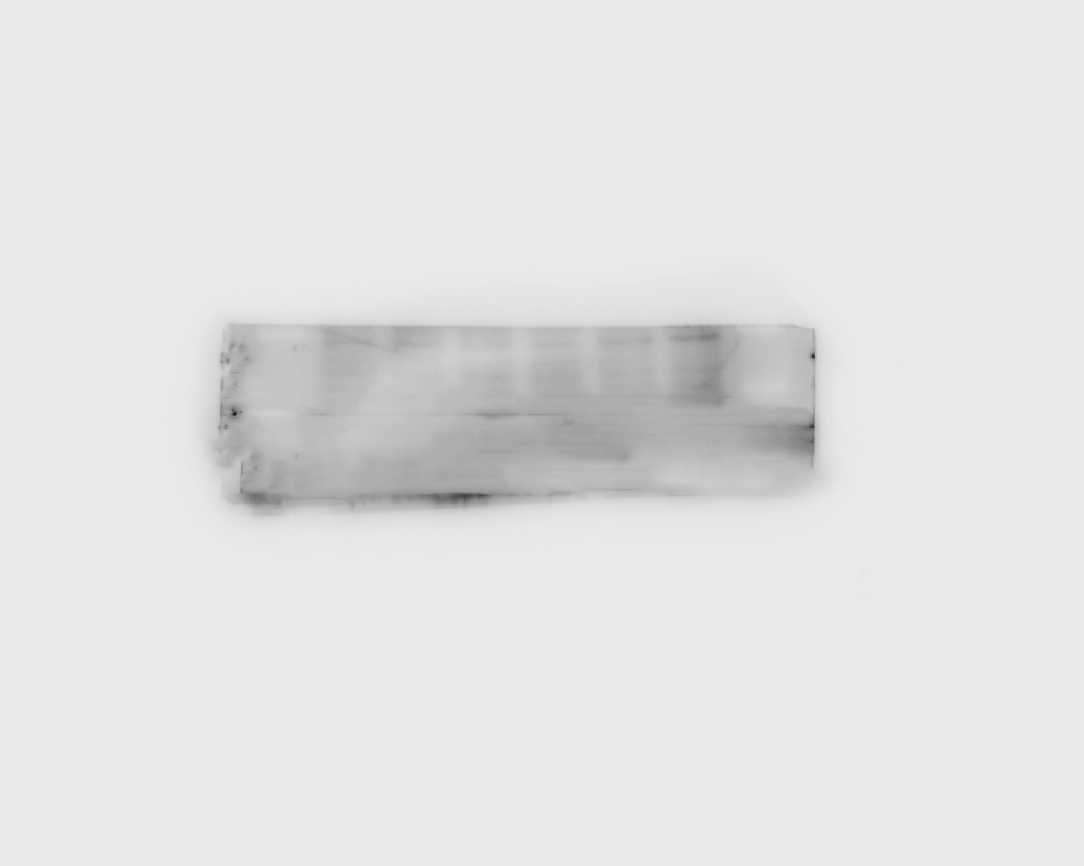


Fig. 3B. Caspase-1 stripe


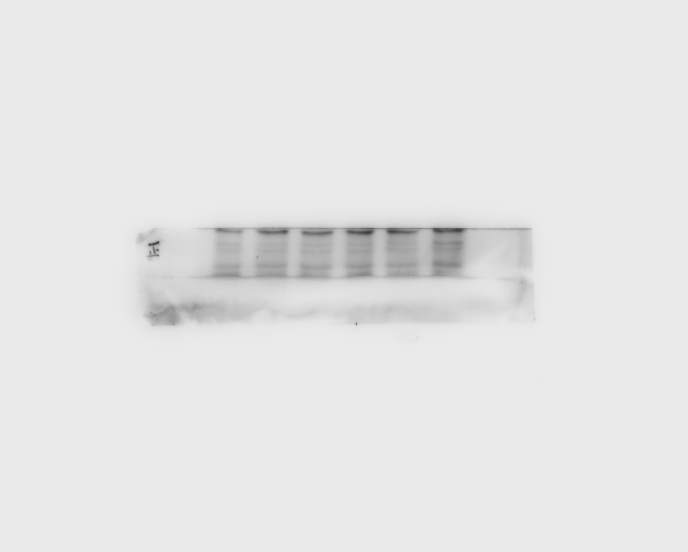


Fig. 3B. GSDMD stripe


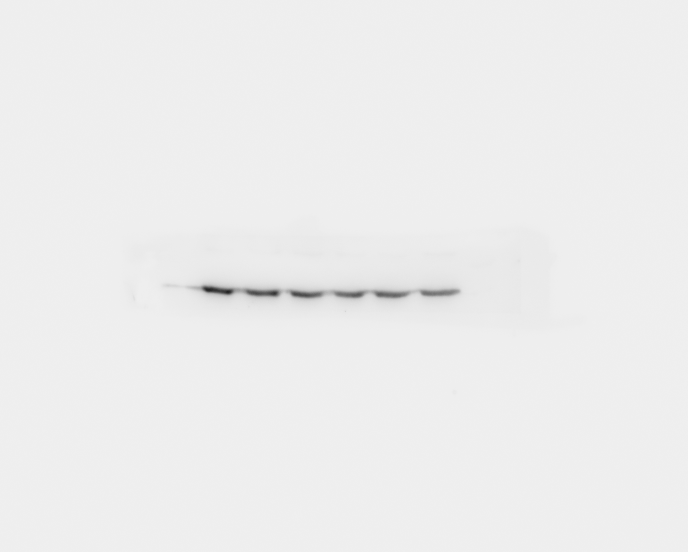


Fig. 3B. β-actin stripe
