# Supplementary material for: MicroRNA-223-3p downregulates the inflammatory response in preeclampsia placenta via targeting NLRP3
Source: BMC Pregnancy Childbirth. 2024 Mar 6;24:175. doi: 10.1186/s12884-024-06371-9 (PMC10918892; doi:10.1186/s12884-024-06371-9)
Supplement: Supplementary file 1 — Supplementary Material 1: Shows the original images of Fig.1B and Fig.3B [file 12884_2024_6371_MOESM1_ESM.docx]

**MicroRNA-223-3p downregulates the inflammatory response in preeclampsia placenta via targeting NLRP3**

Xueqiong Liu^a, b^, Zhiyue Li^a^, Dan Lu^a,^ *

^a^Clinical Medical College of Yangzhou University, Yangzhou 225001, China

^b^The Second Affiliated Hospital of Xuzhou Medical University, Xuzhou 221006, China

Correspondence to: Lu Dan, Email: ludan1968@126.com

In-house Editorial Comments:

We have reviewed the figures provided with the manuscript and must ask that the following editorial requests are addressed during revision:

We note that provided gel images are heavily cropped (Figure 1B, Figure 3B). We ask that as full as possible length gels and blots with membrane edges visible are included in the Supplementary Information file. These images should be the original, unprocessed versions.

**Author response**

Dear Editor, here are our original images, please check:

Fig1B:

Fig3B:
